# The macrophage polarization in *Entamoeba histolytica* infection modulation by the C fragment of the intermediate subunit of Gal/GalNAc-inhibitable lectin

**DOI:** 10.3389/fimmu.2024.1430057

**Published:** 2024-07-19

**Authors:** Dai Dong, Yuhan Zhang, Wenjie Li, Hongze Zhang, Xunjia Cheng, Meng Feng

**Affiliations:** ^1^ Department of Medical Microbiology and Parasitology, School of Basic Medical Sciences, Fudan University, Shanghai, China; ^2^ Women’s Hospital of Nanjing Medical University, Nanjing Women and Children’s Healthcare Hospital, Nanjing, Jiangsu, China

**Keywords:** *Entamoeba histolytica*, macrophage polarization, Gal/GalNAc-inhibitable lectin, immune modulation, host-parasite interactions, inflammatory response

## Abstract

The protozoan parasite *Entamoeba histolytica* is the causative agent of amebiasis, with clinical outcomes ranging from asymptomatic infections to severe invasive diseases. The innate immune system, particularly macrophages, is of paramount importance in resisting the invasion of host tissues and organs by the trophozoites of *E. histolytica*. Parasite-derived pathogenic factors, such as lectins, play a pivotal role in the promotion of macrophage polarization phenotypes that have undergone alteration. Nevertheless, the precise mechanisms by which *E. histolytica* modulates immune polarization remain largely unknown. The current study focused on the immunomodulatory effects of the Igl-C fragment of *E. histolytica* Gal/GalNAc lectin on macrophage polarization. These results demonstrated that Igl-C could induce the secretion of IL-1β, IL-6, and other cytokines, activating a mixed M1/M2 polarization state. M1 polarization of macrophages occurs in the early stages and gradually transitions to M2 polarization in the later stages, which may contribute to the persistence of the infection. Igl-C induces the macrophage M1 phenotype and causes the release of immune effector molecules, including iNOS and cytokines, by activating the NF-κB p65 and JAK-STAT1 transcription factor signaling pathways. Furthermore, Igl-C supports the macrophage M2 phenotype via JAK-STAT3 and IL-4-STAT6 pathways, which activate arginase expression in later stages, contributing to the tissue regeneration and persistence of the parasite. The involvement of distinct signaling pathways in mediating this response highlights the complex interplay between the parasite and the host immune system. These findings enhance our understanding of the Igl-C-mediated pathogenic mechanisms during *E. histolytica* infection.

## 1 Introduction

*Entamoeba histolytica* is an enteric and extracellular protozoan parasite that causes amebiasis, including intestinal and extraintestinal disorders (1). Although 90% of *E. histolytica* infections are asymptomatic, 50 million people are estimated to suffer from invasive amebiasis worldwide, causing approximately 100,000 deaths annually and ranking as the second leading cause of death among protozan parasitic diseases (2–6). When tissue invasion by *E. histolytica* results in rapid development of natural or innate resistance mechanisms, the immune system begins to respond by the action of dendritic cells and macrophages (7). The mucins that line the colonic epithelium serve as important innate defense mechanisms against the invasion of trophozoites into the tissue.

Macrophages play a pivotal role in host resistance to *E. histolytica* trophozoites in various tissues and organs (8). During infection, trophozoites bind to colonic mucins with high affinity via Gal/GalNAc-inhibitable lectins, including the intermediate subunit, to activate macrophages and undergo several metabolic and functional changes, resulting in physiological alterations and inducing different functional phenotypes. In previous studies, we exposed mouse macrophages to trophozoite lysates *in vitro* and observed that the secreted proteins from *E. histolytica* contribute to modulating macrophage polarization balance (9). Furthermore, our studies demonstrated that intrahepatic injection of *E. histolytica* trophozoites in hamsters resulted in the polarization of the macrophage response within the liver tissue (10). Macrophages can be classified into two main categories, M1 and M2, based on their response to external stimuli. M1 macrophages are activated by surface protein-activating stimuli in the microenvironment and are responsible for pro-inflammatory responses, such as phagocytosis and anti-trophozoite activity. In contrast, M2 macrophages are activated by different stimuli and are involved in tissue remodeling and persistent inflammation (11). The polarization process depends on the cytokines produced, the duration of antigen exposure, and pathogen-derived pathogenic molecules. The presence of interferon-γ (IFN-γ) and TNF-α in the microenvironment may facilitate the differentiation of the M1 subset of macrophages, which can present antigens and promote the production of pro-inflammatory cytokines. On the other hand, a microenvironment containing a high concentration of Th2 cytokines, such as Interleukin-4 (IL-4), IL-10, and IL-13, is conducive to the development of M2 macrophages. This macrophage subset exhibits a low antigen presentation capacity and immune regulatory properties, which collectively result in the inhibition of inflammatory responses. This is achieved by the inhibition of T-cell proliferation and activity. Furthermore, M2 macrophages have been implicated in extracellular matrix remodeling and angiogenesis, promoting pathological tissue repair.

Macrophages are well known as innate immune cells with broad and dynamic phenotypic plasticity. At the level of signal transduction and molecular phenotypes, M1 macrophages are characterized by the induction of signal transducer and activator of transcription 1 (STAT1), nuclear factor kappa B (NF-κB) transcription factors, and pro-inflammatory cytokines including TNF-α, IL-1β, IL-6 and IL-12. The M2 macrophages are primarily characterized by the activation of the IL-4Rα/STAT6 and IL-10/STAT3 signaling pathways. IL-4 binds to the cell surface IL-4 receptor α to activate STAT6 phosphorylation. IL-10 induces macrophage STAT3 phosphorylation, and activated STAT3/6 transfers to the nucleus, participating in the regulation of M2 phenotype. STAT6 is the primary transcription factor responsible for regulating M2-related gene during macrophage M2 polarization, including Arg1 (arginase-1) (12, 13). While p-STAT3 is involved in M2 polarization, it also inhibits the activation of NF-κB and expression of p-STAT1, suppressing macrophage M1 polarization and promoting M2 polarization. Stimuli from *E. histolytica* trophozoites or their surface proteins initiate signaling cascades that may induce STAT1, STAT3, STAT6, and NF-κB signaling, ultimately interfering with macrophage phenotypic alteration via epigenetic (re)programming. Nevertheless, the mechanism by which galactose-binding lectins, including the intermediate subunit, induce macrophage polarization remains to be elucidated. Moreover, the biological consequences of such proteins on macrophage differentiation remain elusive.

A previous study reported that the galectin intermediate subunit protein (Igl) of *E. histolytica* plays a pivotal role in the adhesion of trophozoites to host cells and their cytotoxic effect. Immunization of hamsters with natural proteins and recombinant Igl has been shown to protect them from amebic liver abscess (ALA) formation (10). The *E. histolytica* genome contains a multitude of proteins rich in CXXC and CXC motifs (14). The CXXC motif can alter disulfide bonds between the surface proteins of pathogens, leading to the formation, reduction, and rearrangement of these bonds (15). This can lead to antigenic variation, which inhibits the inflammatory response and ultimately causes persistent infection by pathogens. The Igl-C fragment contains the greatest number of CXXC motifs, and the precise mechanism by which Igl-C modulates the immune microenvironment to facilitate *E. histolytica* pathogenesis remains to be elucidated. Consequently, it is crucial to investigate the relationship between Igl-C and macrophage polarization in *E. histolytica*. In this study, we tested the hypothesis that modulation of *E. histolytica* infection by Igl-C promotes macrophage polarization for the first time.

## 2 Materials and methods

### 2.1 Cell culture

The macrophage cell line RAW264.7 (obtained from the American Type Culture Collection (ATCC)) was cultured in Dulbecco’s modified Eagle’s medium (DMEM) (10-013-CV, Corning) supplemented with 20% fetal bovine serum (FBS) (SV30087.02, Hyclone) and 100 U/mL penicillin-streptomycin (Gibco, USA). Cells were maintained in a standard culture environment at 37°C and 5% CO_2_.

### 2.2 Murine IL-4 and recombinant Igl-C *in vitro* stimulation of RAW264.7 cells

Recombinant Igl-C was synthesized using a mammalian expression system (OriGene, USA). The Igl-C coding sequence was amplified and inserted into the PS10001 expression vector, which contained a C-terminal DDK tag. HEK293 cells were transfected with the plasmids and cultured for 48 hours. The cells were then lysed and the soluble fraction containing Igl-C was purified using anti-FLAG affinity chromatography. The purified protein was filtered through a 0.22-μm filter and its purity was confirmed by SDS-PAGE. The RAW264.7 cells were seeded in 24-well plates and allowed to adhere overnight in the absence of serum. The cells were stimulated with recombinant murine IL-4 (214-14, PeproTech) or left untreated for 15 minutes, followed by the addition of LPS or recombinant Igl-C at specified concentrations for 24 or 48 hours. The control samples received only the culture medium. The cells were harvested for RNA and protein isolation and then subjected to downstream analyses, including quantitative PCR, RNA sequencing, and western blotting.

### 2.3 RNA extraction and quantitative RT-PCR

Total RNA was extracted from RAW264.7 cells using the RNeasy Plus Mini Kit (Qiagen, Germany). One microgram of purified RNA was reverse-transcribed into cDNA using the PrimeScript 1st Strand cDNA Synthesis Kit (Takara, Japan). Quantitative real-time PCR was performed using TB Green Premix Ex Taq (Tli RNase H Plus) (Takara) and a 7500 real-time PCR system (Applied Biosystems). Relative expression levels were determined using the comparative Ct (ΔΔCt) method, and transcript abundance was normalized to actin. At least three independent biological replicates were used for each gene. The RT-qPCR primers specific to various mouse genes are listed in **Supplementary Table 1**.

### 2.4 RNA sequencing and differential gene expression analysis

The RAW264.7 cells were stimulated with IL-4 and Igl-C for 24 hours. Then total RNA of RAW264.7 cells was extracted using the RNeasy Plus Mini Kit, with three independent biological replicates for each sample. RNA quality was evaluated by gel electrophoresis and a Qubit fluorometer (Thermo, USA). Starting with 200 ng of total RNA, strand-specific sequencing libraries were constructed using the TruSeq RNA Sample Preparation Kit (Illumina, USA), and sequencing was performed using the Illumina Novaseq 6000 instrument. Raw data were processed using Skewer, and data quality was assessed using FastQC v0.11.2. Clean reads were aligned to the human genome using STAR. Differentially expressed genes between the experimental and control groups were identified using DESeq2. Gene set enrichment analysis was conducted using gene sets from MsigDB to investigate differences between the two groups.

### 2.5 Western blot

Cultured cells were harvested using 20 mM Tris-HCl (pH 7.4) in the presence of protease inhibitor cocktail (Sigma-Aldrich, USA) and phosphatase inhibitor cocktail 2 (Sigma-Aldrich). Protein concentrations were determined using the Pierce BCA Protein Assay Kit (Thermo Fisher Scientific, USA). A total of 20 μg of protein was loaded onto a 10-well pre-cast 4-20% gradient SDS-PAGE gel (Tanon, China) and transferred to PVDF membranes (IPVH00010, Millipore). After blocking with 5% skim milk in TBS-T buffer, the membranes were incubated overnight at 4℃ with primary antibodies targeting actin (1:400, Abcam, USA), NF-κB p65 (1:1000, CST, USA), phospho-NF-κB p65 (1:1000, CST), STAT1 (1:1000, CST), Phospho-STAT1 (1:1000, CST), STAT3 (1:1000, CST), Phospho-STAT3 (1:2000, CST), STAT6 (1:1000, CST) and Phospho-STAT6 (1:1000, CST). Subsequently, the membranes were incubated with horseradish peroxidase (HRP)-conjugated goat anti-rabbit immunoglobulin G (IgG) (1:5000, Abcam) at room temperature for 1 hours. Protein bands were visualized using a Tanon 4600SF imaging system.

### 2.6 Immunofluorescence

RAW264.7 macrophages were seeded on sterile glass coverslips and seeded in 6-well plates at a density of 3 × 10^5^ cells per well. Upon reaching the desired confluence, cells were treated with recombinant Igl-C for either 24 or 48 hours. Subsequently, the cells on the coverslips were fixed with 4% paraformaldehyde for 15 minutes and permeabilized with 0.2% Triton X-100 in phosphate-buffered saline (PBS). After blocking with 3% bovine serum albumin (BSA) in PBS for one hour, the cells were incubated with primary antibodies against phospho-NF-κB p65 (1:1000, CST), Phospho-STAT1 (1:50, CST), Phospho-STAT3 (1:200, CST) and Phospho-STAT6 (1:400, CST) for one hour at room temperature. The secondary antibodies, Alexa Fluor 488 goat anti-rabbit IgG (1:500, Thermo Fisher) or Alexa Fluor 594 goat anti-rabbit IgG (1:500, Thermo Fisher), were incubated with the samples for one hour at room temperature in the dark. Subsequently, coverslips were washed and stained with a solution of DAPI (0.25 mg/mL), DABCO (1.25 mg/mL), and glycerol (10%) for nuclear visualization. The samples were then mounted and sealed for confocal microscopy. Confocal images of cell nucleus were captured on a Leica TCS SP8 Confocal Microscope.

### 2.7 Phosphorylation inhibitor pretreatment and cell viability assay

Before IL-4 and Igl-C stimulation, RAW264.7 cells were pretreated with specific phosphorylation inhibitors targeting key proteins in downstream signaling pathways. The inhibitors used were as follows: Caffeic Acid Phenethyl Ester (CAPE) (T6429, TargetMol), which targets the p65 subunit of NF-κB, was used at a concentration of 10 µM for 1 h. Fludarabine (T1038, TargetMol), an inhibitor of STAT1 activation, was administered at a concentration of 50 µM for a period of one hour. Cryptotanshinone, an inhibitor of STAT3, was used at a concentration of 20 µM for a period of one hour. AS1517499, a selective inhibitor of STAT6, was used at a concentration of 100 nM for a period of half an hour. Following the administration of the inhibitor, the medium was promptly replenished for subsequent stimulation.

Cell viability was evaluated in triplicate using the Cell Counting Kit-8 (CCK-8) (Dojindo, Janpan) in accordance with the manufacturer’s instructions. Cells were seeded into 96-well plates at a density of 7,500 cells/well. Following inhibitor treatment, 10 μL of CCK-8 solution containing 2-(2-methoxy-4-nitrophenyl)-3-(4-nitrophenyl)-5-(2,4-disulfophenyl)-2H-tetrazolium (WST-8) was added to the cells, which were then incubated at 37°C for 2 hours. Optical density (OD) was quantified at 450 nm using a Synergy H1 microplate reader (BioTek, USA). Cell viability was calculated using the following formula:


$$\%Ratio\;of\;viable\;cells=\frac{A450_{sample}-A450_{blank}}{A450_{control}-A450_{blank}}\times 100\%$$

### 2.8 Arginase activity assay

Arginase activity was quantified in triplicate using an Arginase Activity Assay Kit (Sigma-Aldrich). After 24 hours or 48 hours of exposure to various stimuli, approximately 1×10^6^ RAW264.7 cells were lysed in 100 μL lysis buffer containing 10 mM Tris-HCl (pH 7.4), 1 μM pepstatin A (Sigma-Aldrich), 1 μM leupeptin (Sigma-Aldrich), and 0.4% Triton X-100. Subsequently, the lysates were centrifuged, after which 40 μL of the supernatant was added to the sample and blank wells. Fifty microliters of 1 mM urea standard working solution and an equal volume of deionized water were added to each well. Subsequently, 10 μL of 5× substrate buffer containing 8 μL arginase buffer and 2 μL Mn solution was added to each sample well. After thorough mixing, plates were incubated at 37°C for 2 hours. The reaction was stopped by the addition of 200 μL of a pre-prepared urea reagent, followed by an additional 60 minutes of incubation at ambient temperature. OD was measured at a wavelength of 430 nm. Arginase activity was quantified using the following equation:


$$Activity=\frac{A430_{sample}-A430_{blank}}{A430_{standard}-A430_{water}}\times \frac{1mM\times 50\mu L\times 10^{3}}{V\times T}$$

A single unit of arginase activity is defined as the amount of enzyme required to convert 1.0 µmol of L-arginine to ornithine and urea per minute at pH 9.5 and 37°C.

### 2.9 Nitric oxide determination by Griess assay

The indirect assessment of nitric oxide (NO) release was indirectly assessed by measuring the accumulation of nitrite with the Nitrite Assay Kit (Sigma-Aldrich). In brief, cultured RAW264.7 cells were exposed to various stimuli for 24 hours or 48 hours, after which 100 μL of supernatant was collected. Subsequently, 40 μL of each sample was combined with 100 μL of Griess reagent in a 96-well plate, and the final volume was adjusted to 100 μL using deionized water. The reaction mixture was then incubated in the dark at room temperature for 15 minutes. The absorbance of the azo dye was measured at 540 nm using a Synergy H1 microplate reader (BioTek). The nitrite concentration was determined by comparison with a standard curve generated using pure sodium nitrite (NaNO₂), and the assay was conducted at least in triplicate to ensure accuracy.

### 2.10 Statistical analysis

All data are presented as mean ± standard error of the mean (SEM). To determine statistical significance, unpaired two-tailed Student’s *t*-test (or Mann–Whitney test when the data distribution was not normal), one-way ANOVA, or two-way ANOVA with Šídák’s multiple comparisons test was performed using GraphPad Prism 9. Hierarchical clustering was conducted after z-score standardization across all rows, without grouping all heat maps. In all figure panels, n.s., not significant, *P< 0.05, **P< 0.01, and ***P< 0.001.

## 3 Results

### 3.1 Igl-C modulates temporal polarization dynamics in RAW264.7 macrophages

**Figure 1:**
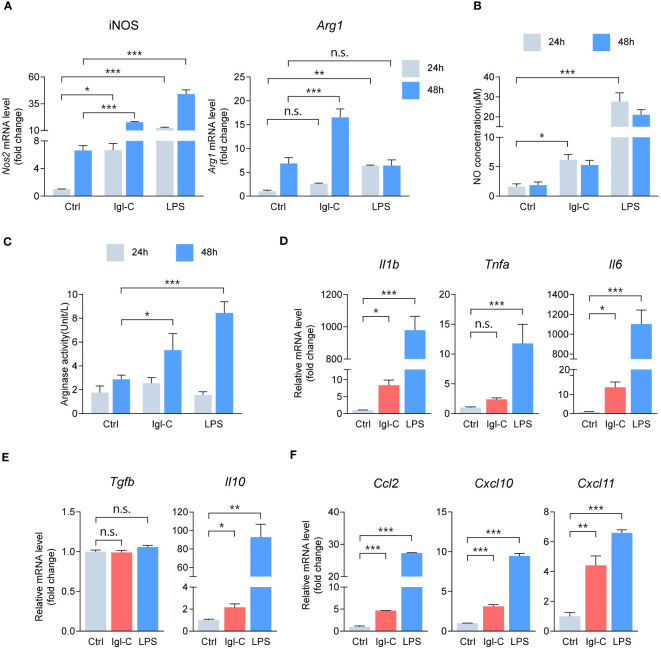
Igl-C modulates RAW264.7 macrophage polarization. **(A)** Gene expression of hallmarks for macrophage polarization in RAW264 cells treated with Igl-C and LPS. n=4. **(B)** Quantification of nitric oxide (NO) concentration via the Griess assay after 24 hours and 48 hours treatment of RAW264.7 cells. n=5. **(C)** Arginase enzymatic activity measured via the colorimetric assay after 24 hours and 48 hours treatment of RAW264.7 cells. n=8. **(D-F)** RT-qPCR detecting expression of pro-inflammatory cytokines **(D)**, anti-inflammatory cytokines **(E)** and chemokines **(F)** in RAW264.7 macrophages after 24 hours treatment. n=3. n.s.=not significant, *P< 0.05, **P< 0.01, ***P< 0.001 by two-way ANOVA followed by Dunnett’s multiple comparisons test for **(A)**, one-way ANOVA followed by Dunnett’s multiple comparisons test for **(B, D–F)**, Kruskal-Wallis test followed by Dunn’s multiple comparisons test for **(C)**.

Induction of inducible nitric oxide synthase (iNOS) is a hallmark of M1 macrophage polarization and plays a pivotal role in the nitrate-nitrite-NO biochemical pathway, ultimately helping to produce nitric oxide. NO functions as a potent effector free radical, contributing to microbial killing and the modulation of pro-inflammatory responses. Moreover, polarization toward the M2 phenotype, particularly the M2a subset, is characterized by the upregulation of Arginase I, which supports tissue remodeling and immunosuppressive functions. To assess the temporal changes in macrophage activation in response to Igl-C treatment, we initially determined the expression of polarization hallmarks in RAW264.7 macrophages exposed to either Igl-C or LPS after 24 and 48 hours. As assessed by RT-qPCR, the Igl-C-treated group exhibited elevated iNOS levels compared with the control group at 24 hours (**Figure 1A**). In contrast, the M2 polarization hallmark, *Arg1*, was upregulated 48 hours after treatment (**Figure 1A**). **Figures 1B, C** illustrate the increase in NO concentration and arginase activity, respectively. These observations align with the temporal gene expression data, indicating a dynamic interplay between M1 and M2 phenotypes in response to Igl-C treatment. Subsequently, the expression of pro- and anti-inflammatory genes was examined following Igl-C treatment for 24 hours. We observed upregulation of the pro-inflammatory molecules IL-1β and IL-6, as well as the anti-inflammatory molecule IL-10 (**Figures 1D, E**). The upregulation of the chemokines *Ccl2*, *Cxcl10*, and *Cxcl11* indicated that Igl-C stimulation activated RAW264.7 macrophages toward the M1 phenotype (**Figure 1F**).

**Figure 2:**
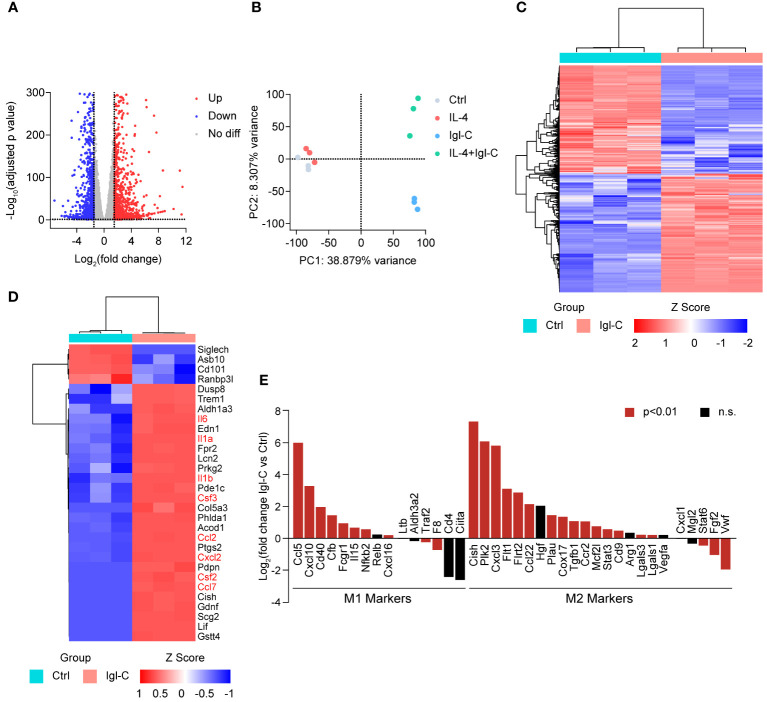
The effect of Igl-C treatment on transcription in RAW264.7 cells. **(A)** Volcano plot of the differentially expressed genes in Igl-C treated versus control RAW264.7 cells. Red and blue dots indicate significantly increased and decreased gene expression, respectively (|log2 foldchange|>1.5 and adjusted p value<0.05). **(B)** PCA analysis of RNA-seq data of control, IL-4, Igl-C and IL-4+Igl-C treated RAW264.7 cells. n=3 biologically replicates. **(C)** Hierarchical clustering heatmap of differentially expressed genes (DEGs) from control and Igl-C treated group. **(D)** Hierarchical clustering heatmap of top 30 DEGs from control and Igl-C treated group. **(E)** Expression pattern of pro-inflammatory and anti-inflammatory marker genes in control and Igl-C treated RAW264.7 cells. Genes showing significant difference (p<0.01) are shown in red.

Based on these findings, we sought to further characterize the broader genetic impact of Igl-C on macrophages through transcriptomic analysis. To identify transcriptional changes in RAW264.7 cells, we next performed RNA-seq on RAW264.7 cells treated with IL-4, Igl-C, or co-treated with IL-4 and Igl-C. A total of 2,239 differentially expressed genes were identified in Igl-C-treated cells compared to controls, with 1,285 genes upregulated and 1,044 genes downregulated (**Figure 2A**). Furthermore, the transcriptional profiles of the control and Igl-C-treated groups were distinct, as revealed by principal component analysis (PCA) and hierarchical clustering (**Figures 2B, C**), indicating significant transcriptional differences between the experimental groups. The heat map in **Figure 2D** shows the top 30 differentially expressed genes. Several cytokines (e.g., *Il6*, *Il1a*, *Il1b*) and chemokines (e.g., *Ccl2*, *Cxcl2*, and *Ccl7*) were significantly upregulated in response to Igl-C stimulation. Intriguingly, we also observed the simultaneous upregulation of genes associated with both M1 and M2 phenotypes (**Figure 2E**), suggesting the coexistence of macrophages exhibiting characteristics of both M1 and M2 polarization states. These results indicate that stimulation with Igl-C has the potential to modulate RAW264.7 cells toward both M1-like and M2-like phenotypes.

### 3.2 Igl-C initiates M1 polarization through NF-κB and STAT1 pathways

**Figure 3:**
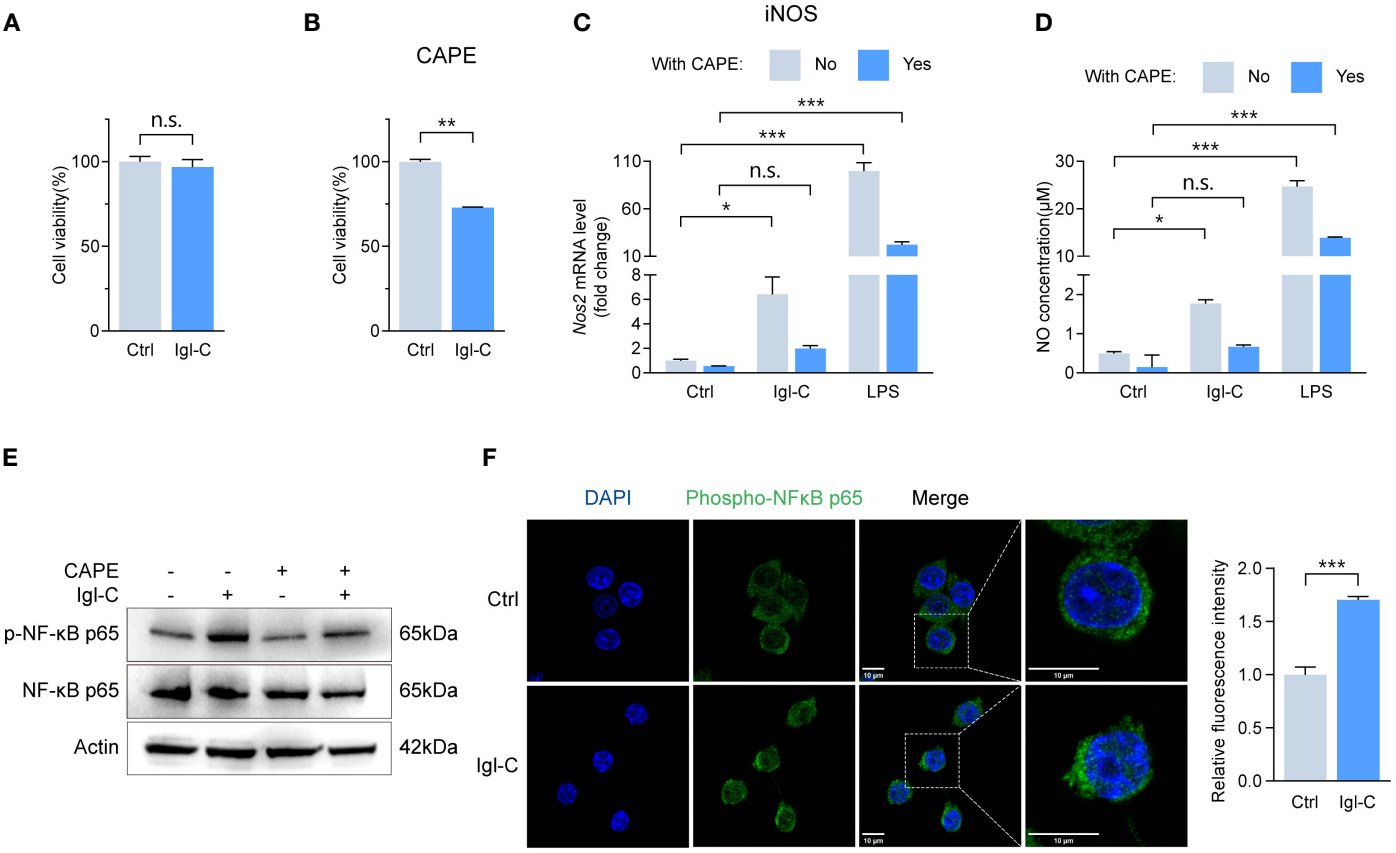
Igl-C initiates M1 polarization through NF-κB phosphorylation. **(A)** Cell viability in response to recombinant Igl-C by CCK-8 assay at 24 hours post-Igl-C stimulation. n=3. **(B)** Cell viability in response to CAPE by CCK-8 assay at 24 hours post-Igl-C stimulation. n=3. **(C)** Gene expression of *Nos2* in RAW264.7 cells treated with Igl-C and LPS, with and without the presence of CAPE at 24 hours post-Igl-C stimulation. n=3. **(D)** Quantification of NO concentration in RAW264.7 cell supernatant via the Griess assay following treatment of Igl-C and LPS, with and without the presence of CAPE at 24 hours post-Igl-C stimulation. n=3. **(E)** Immunoblots of NF-κB p65 and phosphorylated p65 in control and Igl-C treated RAW264.7 cells, with and without the presence of CAPE at 24 hours post-Igl-C stimulation. Actin serves as loading control. **(F)** Immunofluorescence staining and quantification of nuclear fluorescence intensity of phosphorylated NF-κB p65 at 24 hours post-Igl-C stimulation, with nuclei counterstained with DAPI (blue). Scale bar: 10 μm. n.s.=not significant, *P< 0.05, **P< 0.01, ***P< 0.001 by two sided student’s *t* test for **(A, B)**, two-way ANOVA followed by Dunnett’s multiple comparisons test for **(C, D)**.

**Figure 4:**
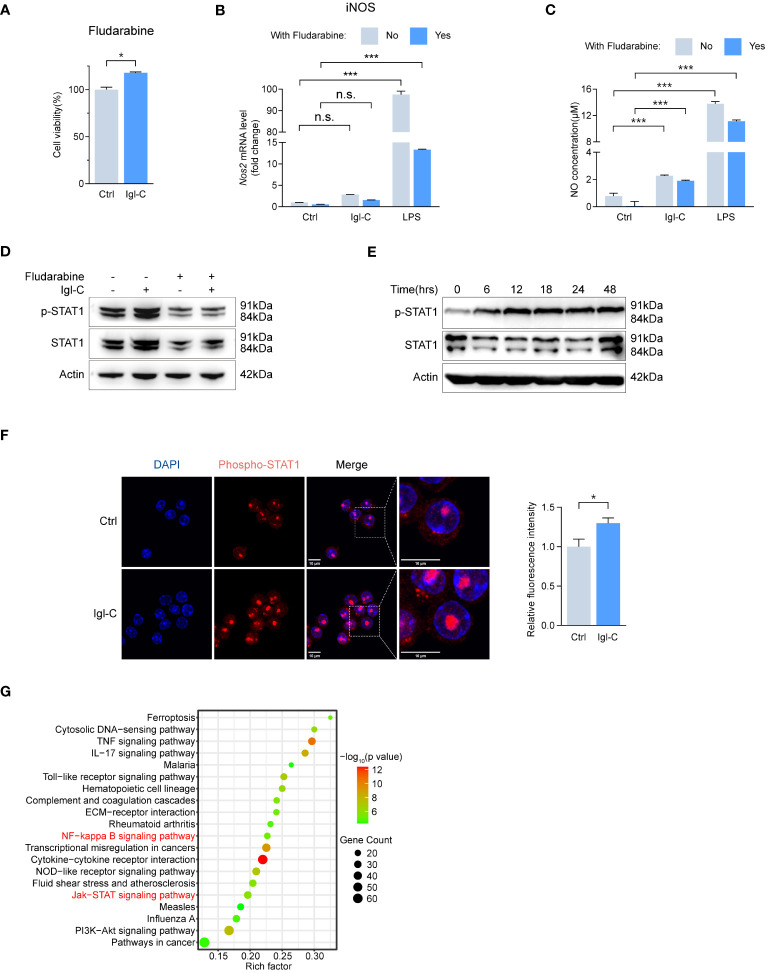
Igl-C initiates M1 polarization through STAT1 phosphorylation. **(A)** Cell viability in response to Fludarabine by CCK-8 assay at 24 hours post-Igl-C stimulation. n=3. **(B)** Gene expression of *Nos2* in RAW264.7 cells treated with Igl-C and LPS, with and without the presence of Fludarabine at 24 hours post-Igl-C stimulation. n=3. **(C)** Quantification of NO concentration in RAW264.7 cell supernatant via the Griess assay following treatment of Igl-C and LPS, with and without the presence of Fludarabine at 24 hours post-Igl-C stimulation. n=3. **(D)** Immunoblots of STAT1 and phosphorylated STAT1 in control and Igl-C treated RAW264.7 cells, with and without the presence of Fludarabine at 24 hours post-Igl-C stimulation. Actin serves as loading control. **(E)** Immunoblots of STAT1 and phosphorylated STAT1 at various time points after Igl-C treatment. Actin serves as loading control. **(F)** Immunofluorescence staining and quantification of nuclear fluorescence intensity of phosphorylated STAT1 at 24 hours post-Igl-C stimulation, with nuclei counterstained with PI (red). Scale bar: 10μm. **(G)** Top 20 upregulated metabolic pathways in Igl-C treated compared to control RAW264.7 cells (using KEGG pathways, presented by enrichment factor) of differentially expressed genes from RNA-seq. n.s.=not significant, *P<0.05, **P<0.01, ***P<0.001 by two sided student’s t test for **(A)**, two-way ANOVA followed by Dunnett’s multiple comparisons test for **(B, C)**.

Signaling through the IFNγ receptor activates the JAK-STAT1 pathway, inducing macrophage polarization toward an “M1-like” state, which is characterized by increased pro-inflammatory activity and resistance to anti-inflammatory factors (16). Furthermore, NF-κB activation promotes M1 polarization, enhancing T-cell immune responses via the upregulation of pro-inflammatory cytokines (17). The induction of M1 polarization is a crucial aspect of the host defense against intracellular pathogens. To ascertain that recombinant Igl-C did not compromise cell viability, we performed a CCK-8 assay (**Figure 3A**). This demonstrated that Igl-C did not elicit cytotoxic effects under the tested conditions. To investigate the signaling pathways involved in the response to Igl-C, cells were pretreated with CAPE, an inhibitor of nuclear transcription factor NF-κB, and fludarabine, a STAT1 activation inhibitor. Pretreatment with CAPE did not alter cell viability (**Figure 3B**). When RAW264.7, cells were pretreated with CAPE, the Igl-C-mediated upregulation of iNOS expression and subsequent NO release was significantly attenuated (**Figures 3C, D**). Western blotting analyses were conducted to corroborate these observations, which revealed elevated phosphorylation of NF-κB p65 in response to Igl-C, which was reduced following CAPE pretreatment (**Figure 3E**). Furthermore, immunofluorescence analysis demonstrated nuclear translocation of phosphorylated NF-κB p65 (**Figure 3F**), illustrating the activation of this transcription factor upon Igl-C treatment. In parallel, Fludarabine-treated cells exhibited a slight increase in viability compared that with of the control (**Figure 4A**). Fludarabine pretreatment only modestly suppressed the Igl-C-induced upregulation of iNOS and was correlated with NO release (**Figures 4B, C**). Consistent with this, western blotting analysis demonstrated a significant increase in STAT1 phosphorylation in the presence of Igl-C, which was reduced by fludarabine pretreatment (**Figure 4D**). The levels of phosphorylated STAT1 exhibited a time-dependent increase during the initial 12 hours, followed by a sustained increase until 48 hours (**Figure 4E**). Immunofluorescence microscopy also demonstrated nuclear translocation of phosphorylated STAT1 following exposure to Igl-C (**Figure 4F**). KEGG pathway analysis, in conjunction with gene set enrichment analysis (GSEA), demonstrated that Igl-C stimulation significantly upregulated the expression of NF-κB target genes, accompanied by a marked enrichment of gene sets associated with the inflammatory response (**Figure 4G** and **Supplementary Figure S1**). Collectively, our data indicate that Igl-C can initiate macrophage polarization toward the M1 phenotype through the involvement of the NF-κB and STAT1 pathways, both of which are essential for the promotion of pro-inflammatory M1 macrophage functions.

### 3.3 Igl-C induces M2 polarization through STAT3 and IL-4 dependent STAT6 pathways

M2 macrophages represent a subset of cells associated with tissue repair, immunoregulation, and anti-inflammatory responses. The polarization of M2 macrophages is regulated by several signaling pathways, including the activation of transcription factors, such as STAT3 and STAT6. Studies have demonstrated that signal transducer and activator of transcription 3 (STAT3) signaling is essential for the anti-inflammatory functions of M2 macrophages, including the suppression of proinflammatory cytokines and promotion of tissue repair. Signal transducer and activator of transcription 6 (STAT6) acts as an upstream mediator pivotal for interleukin-4 (IL-4) and IL-13 signaling, which is essential for M2 polarization (18, 19). The subsequent objective was to ascertain whether STAT3 and STAT6 activation are involved in Igl-C-promoted macrophage M2 polarization.

**Figure 5:**
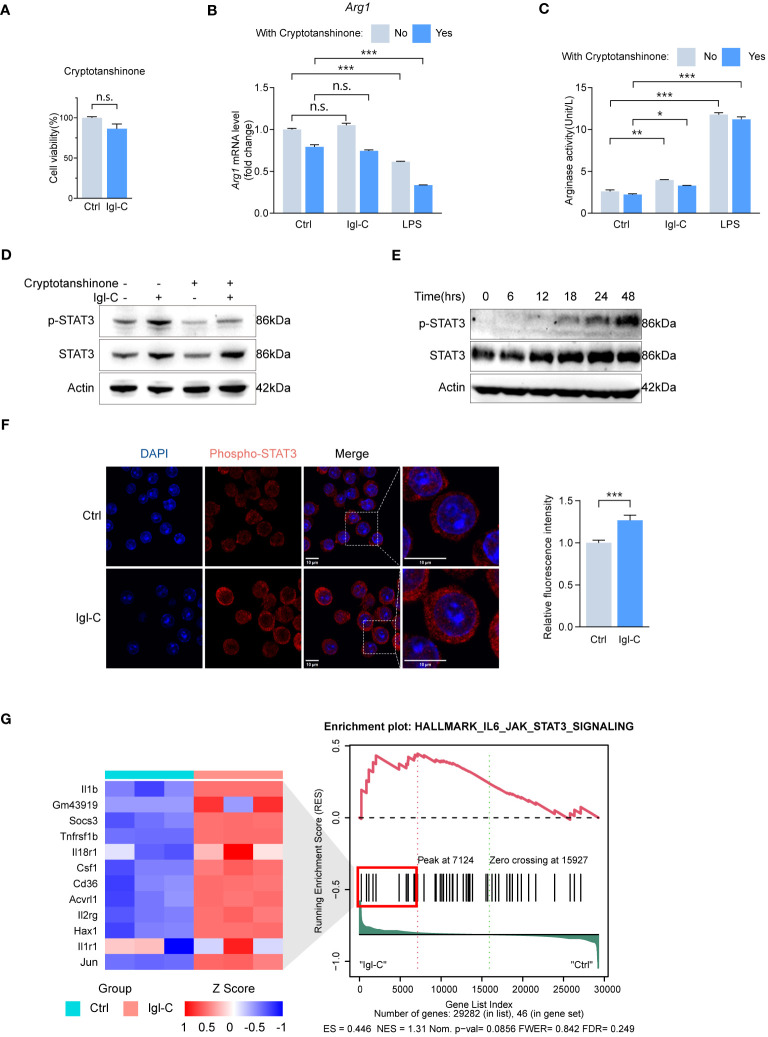
Igl-C induces RAW264.7 macrophage M2-like polarization phenotype through IL-4 depedent STAT6 phosphorylation. **(A)** Cell viability in response to Cryptotanshinone by CCK-8 assay at 48 hours post-Igl-C stimulation. n=3. **(B)** Gene expression of *Arg1* in RAW264.7 cells treated with Igl-C and LPS, with and without the presence of Cryptotanshinone at 48 hours post-Igl-C stimulation. n=3. **(C)** Arginase enzymatic activity measured via the colorimetric assay following a 48h-treatment of RAW264.7 cells using Igl-C and LPS, with and without the presence of Cryptotanshinone at 48 hours post-Igl-C stimulation. n=3. **(D)** Immunoblots of STAT3 and phosphorylated STAT3 in control and Igl-C treated RAW264.7 cells, with and without the presence of Cryptotanshinone at 48 hours post-Igl-C stimulation. Actin serves as loading control. **(E)** Immunoblots of STAT3 and phosphorylated STAT3 at various time points after Igl-C treatment. Actin serves as loading control. **(F)** Immunofluorescence staining and quantification of nuclear fluorescence intensity of phosphorylated STAT3 at 48 hours post-Igl-C stimulation, with nuclei counterstained with DAPI (blue). Scale bar: 10μm. **(G)** Gene set enrichment analysis (GSEA) showing the enrichment of IL6-JAK-STAT3 signaling pathway and hierarchical clustering heatmap of increased genes in Igl-C group. n.s.=not significant, *P< 0.05, **P< 0.01, ***P< 0.001 by two sided student’s *t* test for **(A)**, two-way ANOVA followed by Dunnett’s multiple comparisons test for **(B, C)**.

As assayed by CCK-8, pretreatment with cryptotanshinone, a specific inhibitor of STAT3, did not affect cell survival (**Figure 5A**). Following treatment with cryptotanshinone, there was minimal change in arginase mRNA expression and activity (**Figures 5B, C**). Immunoblotting revealed elevated levels of phosphorylated STAT3 reduced by cryptotanshinone treatment (**Figure 5D**). Phosphorylation of STAT3 was not observed during the initial 12 hours after Igl-C stimulation. However, a gradual increase in phosphorylated STAT3 levels was observed from 18 to 48 hours (**Figure 5E**). Furthermore, immunofluorescence analysis demonstrated nuclear translocation of phosphorylated STAT3 following Igl-C treatment (**Figure 5F**). Pathway enrichment analysis also identified upregulation of IL6-JAK-STAT3 signaling (**Figure 5G**), which supports the involvement of STAT3 phosphorylation in Igl-C-mediated RAW264.7 macrophage M2 polarization.

**Figure 6:**
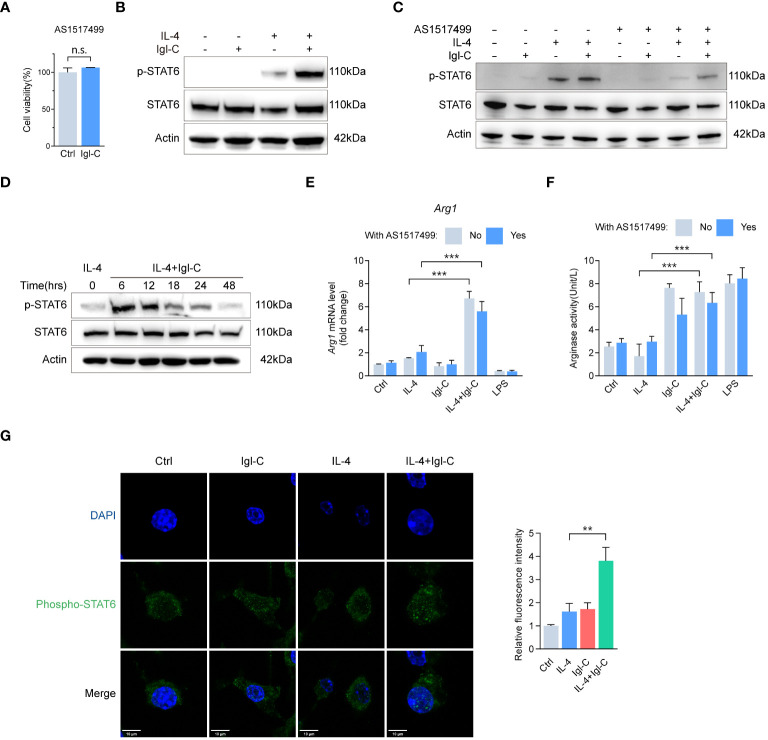
Igl-C induces RAW264.7 macrophage M2-like polarization phenotype through IL-4 depedent STAT6 phosphorylation. **(A)** Cell viability in response to AS1517499 by CCK-8 assay at 48 hours post-Igl-C stimulation. n=3. **(B)** Immunoblots of STAT6 and phosphorylated STAT6 in RAW264.7 cells treated with Igl-C, IL-4, and their combination at 48 hours post-Igl-C stimulation. Actin serves as loading control. **(C)** Immunoblots of STAT6 and phosphorylated STAT6 in RAW264.7 cells treated with Igl-C, IL-4, and their combination, with and without the presence of AS1517499 at 48 hours post-Igl-C stimulation. Actin serves as loading control. **(D)** Gene expression of *Arg1* in RAW264.7 cells treated as indicated, with and without the presence of AS1517499. n=3. **(E)** Arginase enzymatic activity measured via the colorimetric assay following a 48h-treatment of RAW264.7 cells as indicated, with and without the presence of AS1517499. n=3. **(F)** Immunoblots of STAT6 and phosphorylated STAT6 treated with IL-4 and Igl-C in various time points. Actin serves as loading control. **(G)** Immunofluorescence staining and quantification of nuclear fluorescence intensity of phosphorylated STAT6 at 48 hours post-Igl-C stimulation, with nuclei counterstained with DAPI (blue). Scale bar: 10μm. n.s.=not significant, *P<0.05, **P< 0.01, ***P< 0.001 by two sided student’s *t* test for **(A)**, two-way ANOVA followed by Dunnett’s multiple comparisons test for **(E, F)**.

The STAT6-specific inhibitor AS1517499 did not affect cell survival (**Figure 6A**). To extend this, we combined IL-4 and Igl-C treatment to identify differentially expressed genes, because Igl-C alone did not result in increased STAT6 phosphorylation (**Figure 6B**). The RAW264.7 cells were incubated with IL-4 at a concentration of 20 ng/mL for 15 minutes before medium exchange. Samples treated with IL-4 and Igl-C were separated from those treated with IL-4 or Igl-C alone using PCA. In contrast, the IL-4 treatment group exhibited overlapping clusters with the control group, indicating minimal differences in gene expression profiles associated with the treatment. This was corroborated by a volcano plot comparing the two groups, which demonstrated a limited number of differentially expressed genes (**Supplementary Figure S2**). Immunoblot analysis demonstrated that STAT6 phosphorylation levels remained unaltered following Igl-C treatment alone and exhibited a slight increase following a 15-minute pretreatment with IL-4 alone (**Figures 6B, C**). However, Igl-C significantly increased STAT6 phosphorylation in the presence of IL-4, which was inhibited by pretreatment with AS1517499 (**Figure 6C**). Furthermore, we examined the temporal dynamics of phosphorylated STAT6 after IL-4 and Igl-C treatment. Our findings revealed that phosphorylated STAT6 levels peaked 6 hours post-treatment and subsequently declined gradually up to 48 hours (**Figure 6D**), underscoring the transient nature of STAT6 activation. These observations were corroborated by the finding that Igl-C stimulation following IL-4 pretreatment resulted in elevated *Arg1* mRNA level and arginase activity compared to those in the group treated with IL-4 alone (**Figures 6E, F**). Immunofluorescence analysis revealed distinct nuclear localization of phosphorylated STAT6, with Igl-C treatment inducing a marked translocation of phosphorylated STAT6 to the nucleus in the presence of IL-4 (**Figure 6G**), indicating the role of STAT6 in modulating gene expression in response to Igl-C. Finally, our findings demonstrated that both STAT3 and STAT6 contribute to the anti-inflammatory M2-like phenotype of RAW264.7 macrophages in response to Igl-C by enhancing the expression of genes related to immunosuppression and chemotaxis *in vitro*. Importantly, STAT6 activation was found to be IL-4 dependent, underscoring the pivotal role of IL-4 in STAT6-mediated macrophage polarization.

## 4 Discussion

The objective of this study was to investigate the role of the C-terminal fragment of *the E. histolytica* Gal/GalNAc lectin intermediate subunit (Igl-C), a protein with a rich CXXC motif, in the modulation of *E. histolytica*-promoted macrophage polarization. Four aspects of the interaction between Igl-C and macrophages were studied: (I) whether Igl-C could interact with macrophages to induce their polarization, (II) whether Igl-C acts upstream to regulate IL-1β and IL-6 secretion and polarization-related chemokine activation, (III) whether Igl-C activates the NF-κB p65 and STAT1 pathways to induce M1 polarization in macrophages, and IL-10-STAT3 activated by Igl-C induces M2 polarization, and (IV) whether IL-4 is involved in activating STAT6 *in vitro*. Macrophages undergo a transition from M1 to M2 polarization, resulting in the persistence of infection.

This study demonstrated that the Igl-C of *E. histolytica* induces polarization of macrophages and regulates high-output inflammatory responses during disease pathogenesis. Igl-C causes M1 polarization of macrophages in the early stage and gradually transitions to M2 polarization in the later stage, which is one reason for the gradual shift from pro-inflammatory killing effects to persistent infection of host cells during the interaction between trophozoites and host cells. Given that macrophages with M1 polarization secrete pro-inflammatory cytokines, antibacterial agents, and antitumor factors, whereas macrophages with M2 polarization exhibit immune suppression and tissue repair properties (11), the study of macrophage polarization transcriptional modulation may facilitate the identification of trophozoite mechanisms, which could cause persistent infection in the host. This study demonstrates that macrophages elicit rapid pro-inflammatory responses by sensing Igl-C. The innate immune system protects the host from serious invasive infections and promotes tissue damage in amoebic infections via an inflammatory response. The subsequent transition of macrophages to the M2-like phenotype induced by Igl-C in the presence of interleukin 4 (IL-4) demonstrated anti-inflammatory and reparative activities, contributing to the resolution of inflammation and promotion of tissue repair.

Macrophages may exhibit mixed or hybrid phenotypes beyond the two classical polarization phenotypes *in vivo*, depending on the context of inflammation or tissue repair. Macrophage polarization is a dynamic and plastic process influenced by various microenvironmental cues received from the pathogen (20, 21), including cytokines and pathogen-associated molecular patterns (PAMPs) (22–24). Igl, particularly Igl-C, is a specific amoebic molecule that directly stimulates macrophage function. There is evidence that patients with chronic trypanosomiasis have higher levels of serum cytokine M1, and the polarization effect of drugs on M2 can reduce the inflammation and fibrosis of chronic trypanosomiasis (25, 26). Pathogens can persist in infected tissues at low levels for extended periods, and hosts may not exhibit overt clinical symptoms. A strategy employed by pathogens to overcome host restrictions during persistent infection is the manipulation of macrophage polarization (27, 28).

The hypothesis that macrophages induce M2 activation in the host via Igl-C was verified, which suggests that patients with amebiasis are prone to chronic persistent infection. Macrophages that have undergone a shift toward an M2 state and have increased trophozoite persistence were observed to partially depend on Igl-C activity. The principal signaling molecules involved in regulating macrophage polarization include STAT transcription factors and NF-κB transcription activating factors (29, 30). Our experimental data demonstrated that the phosphorylation level of NF-κB p65 in macrophages was significantly increased, whereas the expression of phosphorylated p65 was significantly decreased following treatment with inhibitors. Following pretreatment of the inhibitors, the M1 polarization hallmark iNOS in the experimental group exhibited a notable decline, accompanied by a reduction in the concentration of NO. Immunofluorescence confocal microscopy demonstrated that Igl-C induced the translocation of a substantial quantity of activated p65 from macrophages to the nucleus. Consequently, it was evident that Igl-C induces M1 polarization in macrophages by activating the NF-κB p65 pathway. Following a 24-hour incubation period, macrophages exhibited a significant upregulation in the expression of phosphorylated STAT1 upon exposure to Igl-C. Treatment with a specific inhibitor significantly decreased the expression of phosphorylated STAT1. Furthermore, confocal microscopy demonstrated that Igl-C induced nuclear translocation of a considerable quantity of activated STAT1 in macrophages. Consequently, Igl-C can induce M1 polarization in macrophages by activating the STAT1 pathway. Activation of STAT1 pathway might contribute to inhibit the M2 polarization in the early time, resulting in activation of p-STAT6 pathways preceded the expression of Arginase 1 gene and protein. After 24 hours, Igl-C changed to induce M2 polarization and inhibit the expression of iNOS, resulting in decrease of NO concentration in 48 hours’ time point.

When macrophages were treated with a specific inhibitor and then incubated with Igl-C for 48 hours, the gene expression level of the M2 polarization marker, *Arg1*, and the activity of arginase in the experimental group were significantly decreased. However, the expression level of phosphorylated STAT3 was significantly increased, which should have had the opposite effect after the inhibitor treatment. Furthermore, Igl-C activates STAT3 and translocate it to the nuclei of macrophages in large quantities. The STAT6 is primarily involved in the transduction of the Janus kinase (JAK)-STAT pathway induced by IL-4. Activation of M2-type macrophages depends on STAT6 phosphorylation. In this study, we demonstrated the regulatory role of IL-4-STAT6 signaling in modulating macrophage M2 polarization in response to Igl-C stimulation by enhancing M2-related genes. Consequently, we hypothesized that Igl-C is rich in CXXC motifs, which can alter the disulfide bonds between the surface proteins of pathogens and promote the formation, reduction, and rearrangement of disulfide bonds (15), resulting in antigenic variation, inhibition of the inflammatory response, and ultimately persistent infection of pathogens (10, 31, 32). Single-cell transcriptomics has been used to study the functional diversity changes in macrophages in persistent infection status (33). Factors that influence macrophage heterogeneity in the context of persistent infection have been identified, and the various phenotypes, functional programming, and spatial distributions of these cells have been described. However, further research is necessary to substantiate this hypothesis.

During infection with *E. histolytica*, direct contact between trophozoites and host immune cells induces pro-inflammatory responses through intermolecular interactions. Upon contact with *E. histolytica* Gal/GalNAc lectin, macrophages activate the NF-κB signaling pathway, leading to the release of high-mobility group box 1 (HMGB1) (34). It has been demonstrated that *E. histolytica* Gal/GalNAc lectin induces interactions between trophozoite molecules and macrophages. The macrophage polarization phenotype indicates a delicate balance between the host immune defense and parasites. In the multimodal state of host-parasite interaction, a new equilibrium is established between the host’s immune system and the trophozoites to regulate pathogen proliferation, reduce excessive inflammation, and alleviate tissue damage. A previous study demonstrated that trophozoites invade the host and activate the NF-κB signaling pathway, resulting in the production of high levels of tumor necrosis factor-α (TNF-α), IFN-γ, and nitric oxide, which initiate inflammation (35–37). Studies have indicated that M1 macrophages are regulated by STAT1 and associated with enhanced antigen-presenting capabilities (22, 38, 39). This study elucidates the involvement of NF-κB and JAK-STAT1 pathways in mediating Igl-C-induced macrophage polarization. These pathways are well known for their roles in the transcriptional activation of pro-inflammatory cytokines and in the host’s microbial killing mechanisms. Polarized macrophages exert their biological functions by secreting cytokines and other effector molecules.

M2 macrophages are regulated by STAT3, STAT6, and several other factors. Consequently, IL-4 stimulation facilitates STAT6 translocation to the nucleus, where STAT6 is recruited (13). In our previous study, we observed that IL-4 expression was upregulated in hamster liver tissues inoculated with *E. histolytica* SAW755CR trophozoites (9). In this study, we demonstrated that Igl-C can reprogram macrophages toward an M2-like phenotype and that the presence of exogenous IL-4 reinforces this effect through STAT6 phosphorylation. The STAT6 transcription factor facilitates the expression of genes associated with the M2-like phenotype, including *Arg1*, which acts downstream of the IL-4/IL-13 receptor signaling. During M2 polarization, an increase in *Arg1* activity can cause a reduction in NO production. This metabolic competition can alter the local microenvironment from a pro-inflammatory state to a more anti-inflammatory or tissue-regenerative state. The use of cell lines to study the role of Igl-C in regulating macrophage polarization presents a challenge in deciphering the diverse host-parasite interactions that occur *in vivo*. Nevertheless, further investigation is warranted to elucidate the interactions among Igl-C, NF-κB, and STATs. Further investigation is required to elucidate the Igl-C-induced signaling cascade and its impact on disease pathogenesis and the host immune response.

## 5 Conclusion

**Figure 7:**
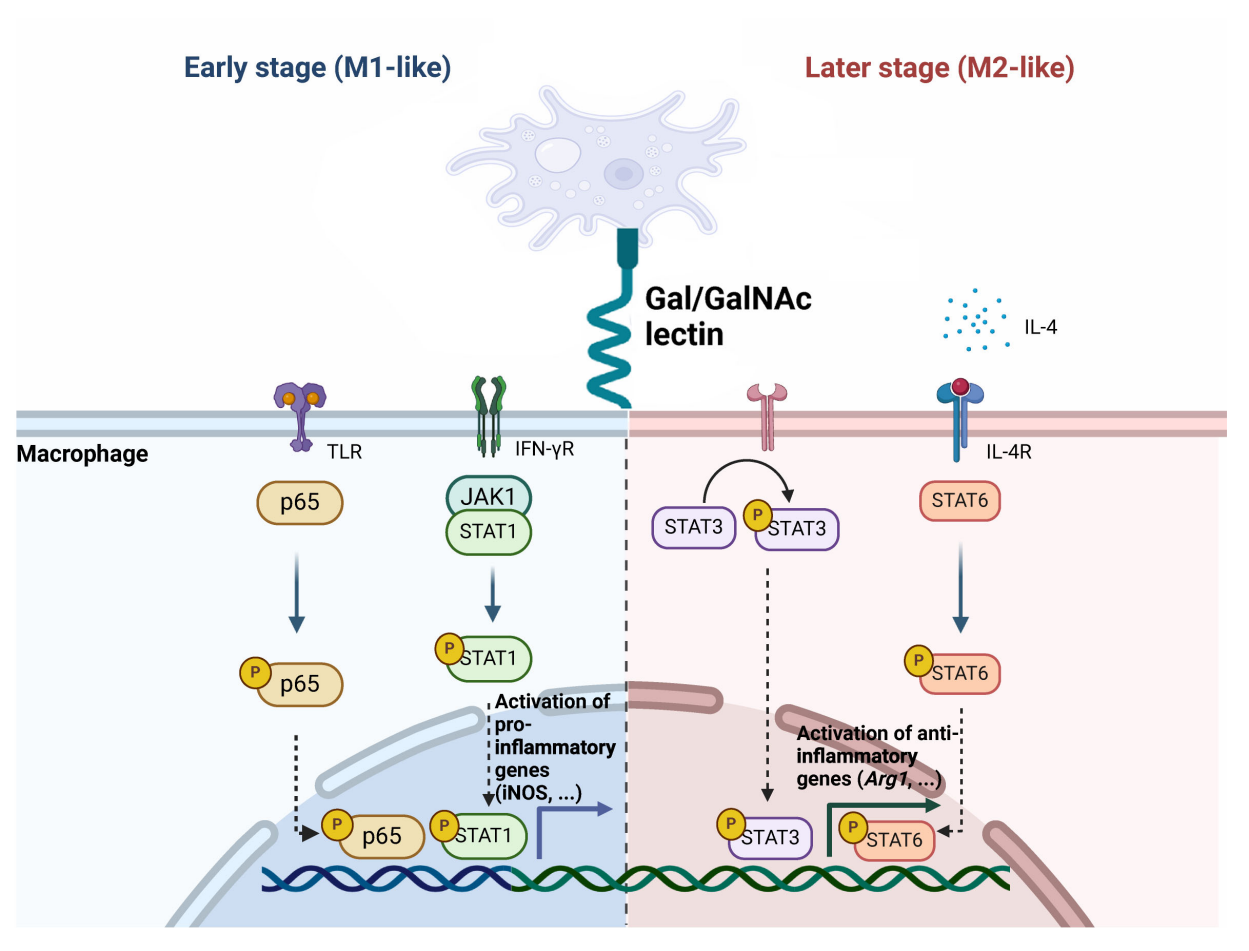
Schematic illustration of the phenotypic transition and various pathways associated with Igl-C-mediated macrophage polarization.

The present study offers novel insights into the immunomodulatory effects of the Igl-C fragment of *the E. histolytica* Gal/GalNAc lectin, which is mediated by macrophage polarization. The capacity of Igl-C to induce a mixed M1/M2 polarization state and the involvement of distinct signaling pathways in mediating this response underscores the intricate interplay between the parasite and the host immune system (**Figure 7**). These findings contribute to our understanding of the pathogenic mechanisms of *E. histolytica*, and may facilitate the development of targeted therapeutic strategies or immunomodulatory approaches against amebiasis.

## Data availability statement

The data presented in the study are deposited in the NCBI’s repository, accession number PRJNA1110832. Further inquiries can be directed to the corresponding author.

## Author contributions

DD: Methodology, Writing – original draft. YZ: Methodology, Writing – original draft. WL: Methodology, Writing – review & editing. HZ: Methodology, Writing – review & editing. XC: Conceptualization, Funding acquisition, Writing – review & editing. MF: Methodology, Writing – review & editing.
