# Supplementary material for: The macrophage polarization in Entamoeba histolytica infection modulation by the C fragment of the intermediate subunit of Gal/GalNAc-inhibitable lectin
Source: Front Immunol. 2024 Jul 19;15:1430057. doi: 10.3389/fimmu.2024.1430057 (PMC11294158; doi:10.3389/fimmu.2024.1430057)
Supplement: Supplementary file 1 [file DataSheet_1.pdf]

Supplementary Material

1 Supplementary Figures and Tables

1.1 Supplementary Tables

Supplementary Table 1. Mouse gene primers used for qPCR in this study.

Gene	Forward(5'-3')	Reverse(5'-3')
Nos2	TCCTGGAGGAAGTGGGCCGAAG	CCTCCACGGGCCCGGTACTC
Arg1	CAGAAGAATGGAAGAGTCAG	CAGATATGCAGGGAGTCACC
Il1b	ACATCAGCACCTCACAAGCAG	TTAGAAACAGTCCAGCCCATAC
Il10	GGACAACATACTGCTAACCGACTC	TCTTGGAGCTTATTAAAATCACTC
Tgfb	GTGCTAATGGTGGACCGCAACAAC	GTGCTAATGGTGGACCGCAACAAC
Tnfa	GTCGTAGCAAACCACCAA	GGCAGCCTTGTCCCTTGA
Il6	TGCCTTCTTGGGACTGAT	TTGCCATTGCACAACTCTTT
Ccl2	AGGTCCCTATGGTGCCAATGT	CGGCAGGATTTTGAGGTCCA
Cxcl10	CCAAGTGCTGCCGTCATTTTC	GGCTCGCAGGGATGATTTCAA
Cxcl11	GGCTTCCTTATGTTCAAACAGGG	GCCGTTACTCGGGTAAATTACA
Actin	CACTGTCGAGTCGCGTCC	TCATCCATGGCGAACTGGTG

1.2 Supplementary Figures

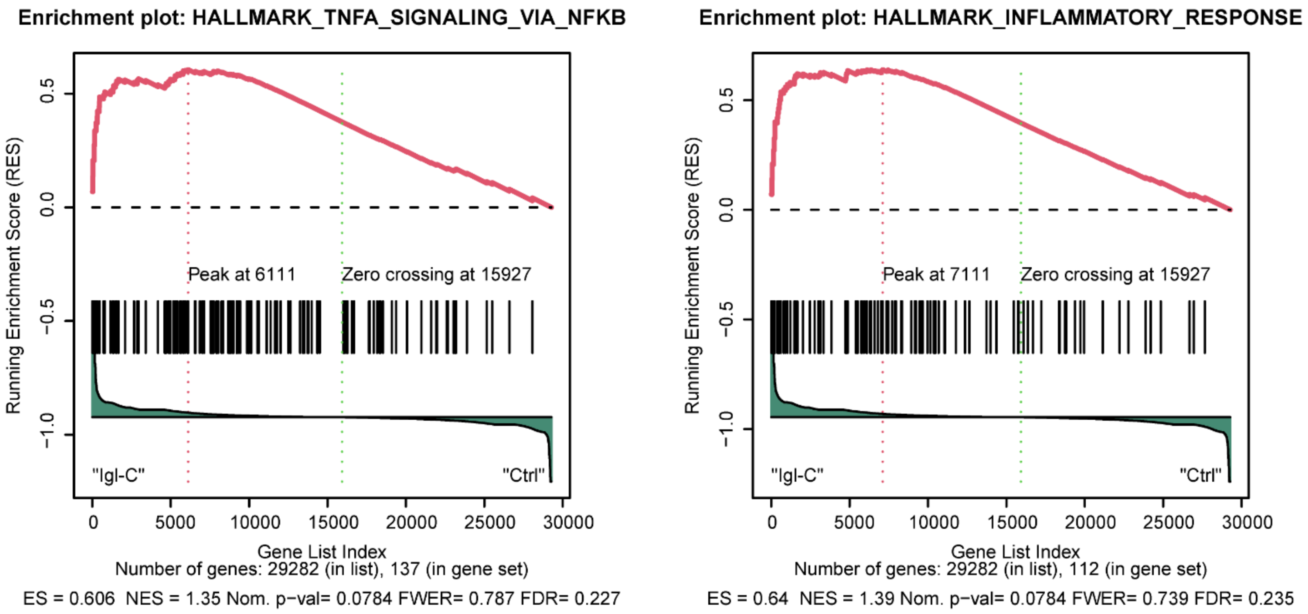

Supplementary Figure 1. Gene set enrichment analysis (GSEA) showing the enrichment of TNF signalling via NF-κB signaling pathway and inflammatory response.

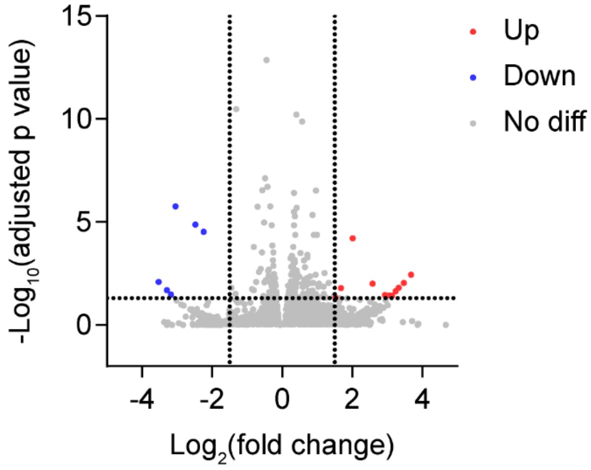

Supplementary Figure 2. Volcano plot of the differentially expressed genes in IL-4 treated versus control RAW264.7 cells. Red and blue dots indicate significantly increased and decreased gene expression, respectively.
